# Identification of drought-responsive genes in roots of upland rice (*Oryza sativa *L)

**DOI:** 10.1186/1471-2164-9-485

**Published:** 2008-10-15

**Authors:** Aline R Rabello, Cléber M Guimarães, Paulo HN Rangel, Felipe R da Silva, Daniela Seixas, Emanuel de Souza, Ana CM Brasileiro, Carlos R Spehar, Márcio E Ferreira, Ângela Mehta

**Affiliations:** 1 Embrapa Recursos Genéticos e Biotecnologia, PqEB Av W5 Norte Final, CEP 70770-900, Brasília, DF, Brazil; 2 Universidade de Brasília, CEP 70910-900, Brasília, DF, Brazil; 3 Embrapa Arroz e Feijão, Rodovia GO-462, km 12 Zona Rural C.P. 179 CEP 75375-000, Santo Antônio de Goiás, GO, Brazil; 4 Universidade Federal do Paraná, Caixa Postal 19046, CEP 81531-990, Curitiba PR, Brazil

## Abstract

**Background:**

Rice (*Oryza sativa *L.) germplasm represents an extraordinary source of genes that control traits of agronomic importance such as drought tolerance. This diversity is the basis for the development of new cultivars better adapted to water restriction conditions, in particular for upland rice, which is grown under rainfall. The analyses of subtractive cDNA libraries and differential protein expression of drought tolerant and susceptible genotypes can contribute to the understanding of the genetic control of water use efficiency in rice.

**Results:**

Two subtractive libraries were constructed using cDNA of drought susceptible and tolerant genotypes submitted to stress against cDNA of well-watered plants. *In silico *analysis revealed 463 reads, which were grouped into 282 clusters. Several genes expressed exclusively in the tolerant or susceptible genotypes were identified. Additionally, proteome analysis of roots from stressed plants was performed and 22 proteins putatively associated to drought tolerance were identified by mass spectrometry.

**Conclusion:**

Several genes and proteins involved in drought-response, as well as genes with no described homologs were identified. Genes exclusively expressed in the tolerant genotype were, in general, related to maintenance of turgor and cell integrity. In contrast, in the susceptible genotype, expression of genes involved in protection against cell damage was not detected. Several protein families identified in the proteomic analysis were not detected in the cDNA analysis. There is an indication that the mechanisms of susceptibility to drought in upland rice are similar to those of lowland varieties.

## Background

Rice (*Oryza sativa *L.) is a cereal of high economic and social value, which is used as a staple food by more than half of the world's population. It is the only cereal which is solely produced for human consumption. The production of rice must increase 20% in the next 15 years in order to keep pace with population growth. One of the main constraints that affect yield in rice production is water deficit. The increasing worldwide water shortage and uneven rainfall distribution limit the use of irrigated agriculture, typical of rice production. Irrigation costs are increasingly high worldwide. There is, therefore, a need to develop rice varieties, which are more efficient in the use of water [1,2]. A major challenge for the research community is the relatively limited progress made so far in improving the drought tolerance of high yielding rice varieties [3].

Rice is a highly diverse species, which can be grown in many types of soil moisture regimes, ranging from aerobic upland to permanently flooded lowland. Although upland rice constitutes a relatively small proportion of the total rice area worldwide, it is the predominant method of rice cultivation in Latin America and West Africa (about 75% and 50% of rice area, respectively) [4]. In Brazil, upland rice responds for approximately 40% of the total rice production. In some areas of the country, upland rice is a subsistence crop planted by farmers who apply limited inputs to their crops. The cultivation of upland rice in marginal areas with low soil fertility and threatened by severe abiotic stresses, such as periods of drought during the cropping season, has a significant impact on rice production [5,6]. Due to exposure to many environmental constraints, some local varieties of the tropical *japonica *rice developed high adaptability to drought stress, hot and dry climatic conditions of regions in Latin America and Africa. Therefore, these varieties may show high levels of water usage efficiency and constitute an excellent material for studying drought tolerance mechanisms in rice. In Brazil, for example, EMBRAPA maintains a germplasm bank enriched with traditional upland rice landraces collected in areas where cultivated rice has been grown since its introduction in the country, centuries ago, and may represent an extraordinary source of genes that control traits of economic importance such as drought tolerance [7].

The determination of the mechanisms directly involved in drought tolerance remains a challenging task since drought is a complex trait that involves several metabolic pathways [3]. The identification and isolation of genes associated with drought tolerance is of major importance in order to better understand this trait and increase the efficiency in developing drought tolerant varieties [8-10]. At the molecular level, the response of roots to water limiting conditions seems to be crucial to trigger drought tolerance mechanisms, since roots are one of the primary sites for stress signal perception in which a signaling mechanism initiates a cascade of gene expression responses to drought. These transcriptional changes can result in successful adaptations leading to stress tolerance by regulating gene expression and signal transduction in the stress response (regulatory proteins) or directly protecting the plant against environmental stress (functional proteins) [11].

Several functional genomic studies of rice have been performed using different approaches such as macro and microarray [12,13], RT-qPCR, SAGE (*Serial Analysis of Gene Expression*), MPSS (*Massive Parallel Signature Sequencing*) and more recently oligoarray using the transcriptome of rice to evaluate responses to abiotic stresses [14]. Proteome analyses have also been increasingly employed to complement genomic studies [15-18], however in a lower rate. Although numerous genes and proteins, which potentially contribute to drought tolerance in rice, have been reported [19-22], most of these studies have focused on lowland rice genotypes. Currently, very little is known about gene and protein expression in upland rice [22-25]. Moreover, most ESTs from drought stressed plants available were obtained from libraries constructed using seedlings [26]. There are very few reports on gene expression of drought-stressed plants in the reproductive stage and using root tissue of plants growing under defined field capacity.

The comprehension of drought responses in upland rice is important for designing breeding strategies to develop varieties more tolerant to water constraints. Recently, the tolerance of ten traditional upland varieties of rice submitted to drought stress has been evaluated as part of an effort to identify new sources of drought tolerance in rice [27]. Concomitantly, the root system of two of the above mentioned upland rice genotypes, characterized as susceptible and tolerant to drought stress, have been analyzed at the reproductive stage using genomic and proteomic approaches. Several genes and proteins were identified, which may play important roles in drought tolerance.

## Methods

### 1. Plant material and phenotypic evaluation

Plants of traditional upland rice (*O. sativa *L. var. *japonica*) varieties were grown on PVC pipe columns (25 cm of diameter; 80 cm of height) filled with fertilized Oxisol under screenhouse conditions [27]. The experimental design was a split-plot design with two watering regimes as main plots, ten traditional upland varieties as subplots and three replications. The watering regimes were (a) control, consisting of a main plot of well-watered plants throughout the experiment, which received 100% reposition of the water lost daily and a minimum soil humidity of -0,025 MPa at 15 cm of depth, and (b) drought stress, which consisted of 50% reposition of the water lost daily from anthesis on. Water reposition was calculated based on daily weighting of columns with a mechanical scale. Twenty-one days after initiating the drought stress treatment (at anthesis), roots of each treatment (control and drought stress) were collected from each rice variety. All root samples were immediately frozen in liquid nitrogen and maintained at -80°C until their use for RNA and protein extractions. At harvest, grain yield and yield components of each genotype were evaluated, including root and shoot dry weight, harvest index, spikelet sterility, grains per panicle and weight of 100 grains. Drought tolerance parameters were estimated based on calculations of drought severity, drought tolerance index and drought susceptibility index [28]. The genotypes submitted to the drought stress showed differences in most of the yield parameters analyzed, which were significantly influenced by the drought severity applied to the experiment [27]. These parameters were then used to classify the genotypes according to their reaction to stress. Among them, two contrasting genotypes for drought stressing conditions were selected for the present study: Prata Ligeiro, as the tolerant, and IRAT20, as the susceptible variety. The RNA and protein analyses proceeded only with root tissue extracted from these two varieties.

### 2. RNA extraction and subtractive library construction

For each genotype, a bulk of approximately 250 mg of plant roots from the three replications were homogenized in liquid nitrogen and total RNA was extracted using the Concert™ Plant RNA Reagent (Invitrogen, USA), according to manufacturer's instructions. This procedure was followed for roots harvested from drought stressed as well as unstressed plants. mRNA was then isolated from total RNA by using PolyATtract mRNA Isolation System (Promega, USA). Quantity and quality of the isolated mRNA was evaluated by spectrophotometry and electrophoresis in agarose gel 1%, respectively.

Isolated mRNAs were used for cDNA synthesis and suppression subtractive hybridization (SSH) library construction by using the PCR Select Subtraction Kit (Clontech, USA). Subtractive hybridizations were performed using cDNA from stressed plant roots (as tester) against cDNA from well-watered unstressed plant roots (as driver) of each genotype, in order to identify genes involved in drought response. The subtractive PCR products obtained were cloned into pGEM T-Easy (Promega, USA) and sequenced in ABI Prism 3700 DNA Analyser (Applied Biosystems Inc., USA). A minimum insert size of 30 bp and at least 20 bp with quality of phred > 20 were considered for the analysis. Sequences were deposited in GenBank under the accession numbers of FG124418 through FG124880 and sequence homologues were identified using the Blast program [29]. An *in silico *subtraction was performed by clustering all sequences from both cDNA libraries according to the methodology described by Telles and da Silva [30], allowing the identification of genes exclusively found in each library.

### 3. Protein extraction and 2-DGE

Total protein was extracted from roots of the drought tolerant (Prata Ligeiro) and susceptible (IRAT20) genotypes according to procedures described by de Mot and Vanderleyden [31] Plant material of the three replications were pooled, pulverized and mixed with extraction buffer (0.7 M sucrose, 0.5 M TrisHCl, 30 mM HCl, 50 mM EDTA, 0.1 M KCl and 40 mM DTT) and phenol (100%) in the same volume (750 μl). Proteins were precipitated with ammonium acetate 0.1 M in methanol, washed with acetone 80% (v/v), dried and stored at -20°C. Protein quantification was performed using the Bradford Reagent (Invitrogen, USA). Isoelectric focusing was conducted using 11-cm immobilized pH gradient (IPG) strips with a pH range of 4–7 and a Multiphor II electrophoresis system (GE). Strips containing approximately 220 μg of protein were rehydrated with 2% (v/v) CHAPS, 8 M urea, 7 mg dithiothreitol (DTT) and 2% IPG buffer. Second dimension analysis was performed in 10% gels by SDS-PAGE as described by Laemmli [32] and at least five replications of each genotype were performed. Protein spots were visualized after silver [33] or Comassie blue staining.

### 4. Image analysis

The 2D gel images were evaluated using the Platinum software (GE Healthcare, UK) and three high quality gels obtained for both genotypes were analyzed. First, a calibration with a grey scale was performed to transform grey levels into OD values for each pixel (px) of the gel image. The wizard detection method proposed by the software was used to detect the spots with the following parameters: 15 px for estimated spot size, 50 px for minimum spot size and a spot contrast enhancement of 75%. Automatically detected spots were checked and some of them were manually added or removed. Following the detection procedure, the normalization step was carried out to attribute a common spot identity for the same spots derived from different images utilizing the reference gel construct and automatically matching options. A synthetic gel from each genotype was constructed by using the mean value of volume percentage of each protein spot present in the three replicates, according to the Platinum software's (GE Healthcare, UK) instructions. The two obtained synthetic gels were then overlapped using the molecular marker as well as several protein spots present in both profiles as landmarks. The overlapped images were based on landmark spots showing same pI and Mw.

### 5. Trypsin digestion and mass spectrometry analysis

Protein spots were excised manually from 2D gels and in-gel digested with sequencing grade trypsin (Promega, Madison, WI) according to Schevchenko et al. [34]. Briefly, each protein spot was placed in a 0.5 mL polypropylene (Eppendorf) tube and destained by washing 5–8 times with 200 μL of 50% (v/v) acetonitrile/10 mM ammonium bicarbonate solution. The gel pieces were subsequently dehydrated by washing with 200 μL of 100% acetonitrile and completely dried in a Speedvac concentrator. Ten microliters of 50 mM ammonium bicarbonate/10% (v/v) acetonitrile solution containing 100 ng of trypsin were added, and the sample incubated at 37°C for 16 h. Aliquots of each tryptic digest (1 μL) were mixed with a saturated solution of α-cyano-4-hydroxycinnamic acid, spotted onto a MALDI target plate, and allowed to air dry.

Mass spectra were acquired using a MALDI-TOF/TOF Autoflex II spectrometer (Bruker Daltonics, Bremen, Germany) operating at a laser frequency of 50 Hz. MS analysis were performed in a positive ion reflection mode. Voltage parameters were set as IS1 19 kV, IS2 16.8 kV, Lens 8 kV, Reflector 20 kV, Reflector2 9.54 kV. The delay time was 70 ns and acquisition mass range 700–3200 Da. External calibration was performed using a peptide mix contaning ACTH (1–24), ACTH (18–39), Somatostatin, Angiotensin I and Angiotensin II, all from Sigma. MS/MS analysis were performed in a positive ion LIFT reflection mode. Voltage parameters used were IS1 6 kV, IS2 5.3 kV, lens 3.15 kV, Reflector 23.5 kV, Reflector2 9.7 kV, LIFT1 19 kV and LIFT2 4 kV. The delay time was set as zero and acquisition mass range 40–2400 Da.

Peak lists were generated using the FlexAnalysis 3.0 software (Bruker Daltonics). The sophisticated numerical annotation procedure (SNAP) algorithm was used to detect the monoisotopic peak values, with a quality factor threshold of 30 and 6 as S/N threshold. Database searches were performed in February 2008 using the MASCOT search engine (Matrix Science, UK) with the NCBInr protein database and *Oryza sativa *taxonomy. The mass tolerance was 100 ppm and one missed cleavage was allowed. Carbamidomethylation of cysteines, oxidation of methionine, and acrylamide-modified cysteines were considered for PMF searches. For accepting the identification, the cutoff value for the Probability Based Mowse score calculated by MASCOT (at *p *< 0.05) was used. For MS/MS data, the peptide mass tolerance was 0.5 Da, MS/MS ion mass tolerance at 0.5 Da, allowance of 1 missed cleavage, and charge state +1. When the pI and MW of matched proteins were not available, these values were calculated using ExPASy Compute pI/Mw tool .

## Results and discussion

### 1. Experimental design and sampling

Plants were submitted to drought stress after anthesis for twenty-one days. Flowering is the period in which the plant is most sensitive to water deficit and several tolerance mechanisms need to be activated at this stage in order to guarantee grain filling and production [6]. During root sampling, a clear visual difference in Prata Ligeiro and IRAT20 plants could be observed. An intense leaf rolling was noticed in the susceptible genotype as opposed to the tolerant. In addition, a more pronounced aerial biomass loss could be visualized in IRAT20. At harvest, yield and yield component parameters were measured [27]. The variety IRAT20, a high yielding variety under irrigated controlled conditions, showed a 51% reduction in grain yield when submitted to drought stress. On the other hand, Prata Ligeiro, a low yielding variety under well watering conditions, had a 23% reduction in grain yield under drought stress. The drought susceptibility index based on yield was estimated as 0.73 for Prata Ligeiro (tolerant) and 1.57 for IRAT20 (susceptible).

Collected roots of both genotypes were then used for cDNA library construction and proteome studies. In the cDNA library study, stressed plants were contrasted with well-watered plants, whereas in the proteome analysis, stressed plants from both genotypes were compared.

Water reposition, based on the evapotranspiration rate, has been used to determine an impartial and consistent response of plants to drought stress, during long periods of drought in the soil [35]. Several studies have tried to define the critical limit of water in the soil after which crop development and production are significantly affected [36]. According to Rosenthal *et al*. [37], the symptoms of water deficit occur when water availability is around 50% of the field capacity.

The response of plants to drought stress is also dependent on the extension and rate of water loss [38]. Fukai *et al*. [39] reported that when a rapid water deficit occurs, the morpho-physiological mechanisms are severely affected. When the deficit is prolonged for a few days, plants are allowed to adapt to the stress, enabling the identification of variability in drought tolerance within different genotypes, since plants can respond differently to the same stress condition [38]. Therefore, the sampling time used in this study (21 days of drought stress) may have allowed the analysis of adaptive responses of the plant to tolerate water deficit.

Several studies reported the response of rice seedlings to drought stress [13,26,40] however, little attention has been given to the expression of genes in water-stressed plants at the reproductive stage (flowering, grain filling) in which a higher yield impact is observed [6].

### 2. cDNA library analysis

Roots are one of the primary sites responsive to restrictive conditions of water availability and, as a result, synthesize chemical signals for a rapid response of the plant to drought stress [41]. This occurs since the response in leaves must be stimulated rapidly to avoid irreversible damage to the photosynthetic machinery. In this work, two subtractive cDNA libraries were constructed using mRNA from roots of tolerant and susceptible upland rice genotypes subtracted from their respective unstressed well-watered controls. The subtracted PCR products obtained after primary and secondary PCR ranged from 0,1 – 1,5 kb.

The SSH libraries of the tolerant (Prata Ligeiro) and susceptible (IRAT20) genotypes were concluded with a novelty index of 66% and 55%, respectively. The general analysis of the two libraries revealed a total of 463 valid sequences (230 from Prata Ligeiro and 233 from IRAT20) and the average fragment size was of 300 bp. Several genes commonly expressed in both genotypes were identified and are probably not directly involved in drought tolerance.

In order to determine the genes exclusively expressed in the tolerant and susceptible genotypes, an *in silico *subtraction was performed using sequences of both libraries. The results for the *in silico *subtraction revealed that the 463 sequences represented 282 different transcripts: 127 were found in both genotypes, 84 were exclusively expressed in the Prata Ligeiro library (Table 1) and 71 were observed only in the IRAT20 library (Table 2).

**Table 1:** Genes detected exclusively in roots of the tolerant genotype (Prata Ligeiro) SSH library

**Encoded protein**	**Homologous organism**	**Accession number**
**Proteins of known function**		
Glutamate-1-semialdehyde 2,1 aminomutase	*Oryza sativa*	NM_001068872
Metallothionein-like protein	*Oryza sativa*	NM_001056317
Malate dehydrogenase	*Oryza sativa*	NM_001062924
Methionine sulfoxide reductase A	*Oryza sativa*	NM_001063272.1
Phosphatidylinosytol 3 and 4 kinase	*Oryza sativa*	NM_001060732
Ubiquitin-conjugating enzyme	*Oryza sativa*	NM_001048429
Nuclear protein SET domain containing protein	*Oryza sativa*	NM_001067672
Splicing factor 3B subunit 5-like protein	*Oryza sativa*	dbj|BAD10044.1|
PEP carboxikinase	*Oryza sativa*	gb|ABF95034.1|
Putative malate dehydrogenase	*Oryza sativa*	gb|AAT69584.1|
Eukaryotic translation initiation factor 5A-2 (eIF-5A) (eIF-4D)	*Oryza sativa*	NC_008405
Metallothionein-like protein type 1	*Oryza sativa*	NP_001068544.1
ADP glucose pyrophosphorylase	*Oryza sativa*	EF122437
CBL-interacting protein kinase 1	*Oryza sativa*	NM_001049327
ADP-ribosylation factor	*Oryza sativa*	NM_001051134
DSS1/SEM1 family protein	*Oryza sativa*	NC_008394
Ankyrin repeat containing protein	*Oryza sativa*	NM_001054582
Pathogenesis-related transcriptional factor and ERF domain containing protein	*Oryza sativa*	NC_008402
E-class P450, group I family protein	*Oryza sativa*	NM_001074239
FAR1 domain containing protein	*Oryza sativa*	NM_001057341
Tubulin alpha-1 chain	*Oryza sativa*	NM_001074145
Putative ubiquitin conjugating enzyme	*Oryza sativa*	dbj|BAB89662.1|
DEAD/DEAH box helicase domain containing protein	*Oryza sativa*	NM_001069156
Putative pollen specific protein C13 precursor	*Oryza sativa*	gb|AAM08621.1|
IQ calmodulin-binding	*Oryza sativa*	NM_001061046
HAD superfamily hydrolase 5' nucleotidase protein	*Oryza sativa*	NM_001057956
SAM biding motif domain containing protein	*Oryza sativa*	NM_001070787
Peptidase aspartic family protein	*Oryza sativa*	NM_001063168
Nonaspanin (TM9SF) family protein	*Oryza sativa*	NM_001056027
Ethylene responsive element binding factor 5	*Oryza sativa*	NM_001063579
TMS membrane protein	*Oryza sativa*	NM_001054899
Heat shock protein DnaJ family protein	*Oryza sativa*	NM_001060020
Ferredoxin III, chloroplast precursor (Fd III)	*Oryza sativa*	NC_008396
Anther ethylene-upregulated protein ER1 (Fragment)	*Oryza sativa*	NM_001055765
Chaperone protein DNA-J-related like	*Oryza sativa*	dbj|BAD27799.1|
Isoflavone reductase family protein	*Oryza sativa*	NM_001068997
U box domain containing protein	*Oryza sativa*	NM_001071339
Ribossomal protein L	*Curculio glandium*	AM049038
Short chain dehydrogenase tic32	*Oryza sativa*	NM_001048577
Arabinogalactan protein	*Oryza sativa*	NC_008394
Ribonuclease T2 family protein	*Oryza sativa*	NM_001070328
HvB12D protein (B12Dg1 protein)	*Oryza sativa*	NM_001063815
Respiratory burst oxidase homolog	*Oryza sativa*	NM_001049555
Phosphatidylinositol-4-phosphate 5-kinase family protein	*Oryza sativa*	NM_001068386
Nodulin-like	*Oryza sativa*	NM_001070322
Cathepsin B-like cysteine protease form 2	*Ixodes ricinus*	gb|ABO26563.1|
Cathepsin L-like cysteine proteinase precursor	*Acanthoscelides obtectus*	gb|AAQ22984.1|
Calcium-transporting ATPase/calmodulin binding	*Arabidopsis thaliana*	NP_188931.1
Myb, DNA biding domain containing protein	*Oryza sativa*	NM_001062445
TGA-type basic leucine zipper protein	*Phaseolus vulgaris*	gb|AF402607.1|
Tocopherol O-methyltransferase, choroplast precursor	*Oryza sativa*	NM_001054379
ATP-dependent Clp protease ATPbiding subunit Clpx-like mitochondrial precursor	*Oryza sativa*	dbj|BAD15818.1|
HvB12D protein (B12Dg1 protein)	*Oryza sativa*	NM_001063815
Uncharacterized protein family containing protein	*Oryza sativa*	gb|ABA91393.1|

**Protein of unknown function**		
Protein of unknown function	*Oryza sativa*	NC_008397
Protein of unknown function	*Oryza sativa*	NC_008403
Unknow function	*Oryza sativa*	NM_001067277
Hypothetical protein	*Oryza sativa*	AP008208
Hypothetical protein	*Oryza sativa*	gb|EAY93896.1|
Conserved hypothetical protein	*Oryza sativa*	NM_001065538
Hypothetical protein	*Oryza sativa*	gb|EAY84091.1|
Hypothetical protein	*Oryza sativa*	CT836006
Hypothetical protein	*Oryza sativa*	NC_008394.1
Hypothetical protein	*Oryza sativa*	NC_008394.1
Hypothetical protein	*Oryza sativa*	AP008208
Hypothetical protein	*Oryza sativa*	NM_001057688
Hypothetical protein	*Oryza sativa*	NM_001066910
Hypothetical protein	*Oryza sativa*	NM_001053573
Hypothetical protein	*Oryza sativa*	CT829595
Hypothetical protein	*Oryza sativa*	CT834076

**Table 2:** Genes detected exclusively in roots of the susceptible genotype (IRAT20) SSH library

**Encoded protein**	**Homologous organism**	**Accession number**
**Proteins of known function**		
T complex 11 family protein	*Oryza sativa*	NM_001059402
Protein kinase domain containing protein	*Oryza sativa*	NM_001071926
Protein disulphide isomerase family protein	*Oryza sativa*	AP008208
TPR-like domain containing protein	*Oryza sativa*	NM_001058028
Protein kinase	*Oryza sativa*	NM_001074788
Pinoresinol-lariciresinol reductase TH1	*Oryza sativa*	NM_001073059
Smr protein; MutS2 c- terminal domain containing protein	*Oryza sativa*	NM_001048992
SIPL protein (Membrane-type 1 matrix metalloproteinase cytoplasmic tail binding protein-1)	*Oryza sativa*	NM_001055581
Similar to CG 9092- PA	*Tribolium castanum*	XP_967647.1
Putative ATP-dependent Clp protease ATP-binding subunit ClpX1 (CLPX)	*Oryza sativa*	dbj|BAD15818.1|
Cytocrome P450 family protein	*Oryza sativa*	NM_001071591
Preprotein translocase subunit sec Y, chloroplast precursor	*Oryza sativa*	NM_001067916
Vacuolar H+ pyrophosphatase	*Oryza sativa*	NM_001063501
Similar to UPF 0139 protein CGI-140	*Tribolium castaneum*	XP_971064.1|
60 kDa inner membrane insertion protein family protein	*Oryza sativa*	NM_001055291
Glyceraldehyde-3-phosphate dehydrogenase (Fragment)	*Oryza sativa*	NM_001055382
Similar to splicing coativator subunit SRm 300	*Monodelphis domestica*	XP_001371550.1|
Cysteine synthase, mitocondrial precursor	*Oryza sativa*	NM_001052112
TPR-like domain containing protein	*Oryza sativa*	NM_001056953
HCO3-transporter	*Oryza sativa*	NM_001073581
Banched chain amino-acid aminotransferase-like protein 3	*Oryza sativa*	NM_001049072
Beta tubulin (fragment)	*Oryza sativa*	NM_001049296
HAT dimerisation domain containing protein	*Oryza sativa*	NC_008402
Urease accessory protein G	*Oryza sativa*	NM_001062872
Glycoside hydrolase, family 47 protein	*Oryza sativa*	NM_001054615
WRKY transcription factor 82	*Oryza sativa*	DQ298186
Tubby family protein	*Oryza sativa*	NM_001062568
Ribosomal protein L41 family protein	*Oryza sativa*	NC_008400
Granule-bound starch synthase I, chloroplast precursor	*Oryza sativa*	NM_001065985
Putative RNA polymerase I transcription factor RRN3	*Oryza sativa*	dbj|BAD45608.1|
Aconitate hydratase, cytoplasmic (Citrate hydro-lyase) (Aconitase)	*Oryza sativa*	NM_001055433
Short chain alcohol dehydrogenase-like	*Oryza sativa*	NM_001056212
Putative ubiquitin-conjugating enzyme E2	*Oryza sativa*	dbj|BAD25096.1|
Peptidase s26A signal peptidase I family protein	*Oryza sativa*	NM_001074823

**Protein of unknown function**		
Unknown protein	*Oryza sativa*	NM_001068742
Hypothetical protein	*Oryza sativa*	AC119292
Hypothetical protein	*Oryza sativa*	AP008208
Hypothetical protein	*Oryza sativa*	AK243578
Hypothetical protein	*Oryza sativa*	NC_008395.1
Hypothetical protein	*Oryza sativa*	AP008208
Hypothetical protein	*Oryza sativa*	NM_001057104
Hypothetical protein	*Oryza sativa*	NC_008395
Hypothetical protein	*Oryza sativa*	NM_001074804
Hypothetical protein	*Oryza sativa*	NM_001057688
Hypothetical protein	*Oryza sativa*	NC_008401.1
Hypothetical protein	*Oryza sativa*	NC_008395.1
Hypothetical protein	*Oryza sativa*	CR855113
Hypothetical protein	*Oryza sativa*	AC145477
Hypothetical protein	*Oryza sativa*	AC092556
Hypothetical protein	*Oryza sativa*	AK242616
Hypothetical protein	*Oryza sativa*	AP008209
Hypothetical protein	*Oryza sativa*	NC_008398.1
Hypothetical protein	*Oryza sativa*	AC099401
Hypothetical protein	*Oryza sativa*	NM_001050487
Hypothetical protein	*Oryza sativa*	CT831698
Hypothetical protein	*Oryza sativa*	CT828847
Hypothetical protein	*Oryza sativa*	CT832865

#### 2.1. Putative drought-tolerance genes identified in Prata Ligeiro

Drought tolerance is a complex trait and involves mechanisms that act in isolation or combined to avoid or tolerate periods of water deficit. It is expected that genotypes responding differently to drought stress show differences in gene expression, and that a portion of the differences is related to drought tolerance. Therefore, the analysis of the genes found exclusively in the tolerant genotype is of interest to identify genes associated with water usage efficiency.

Among the 84 transcripts uniquely reported in the tolerant genotype, 14 did not present known homologs (no hits) and 17 showed similarities to proteins with unknown function (hypothetical proteins). Three sequences showed similarity to non-plant proteins and probably represent contaminating sequences (Table 1). The other transcripts showed similarity to several proteins previously reported as associated to drought stress and some of them are discussed below.

Genes involved in signaling routes were exclusively identified in Prata Ligeiro and include serine/threonine kinase, ethylene-responsive factor and calcium-transporting ATPase/calmodulin binding sequences. Serine/threonine kinases are Ca^2+ ^dependent proteins kinase (CDPKs), involved in the phosphorylation cascade of proteins. Several studies have shown that CDPKs are induced or activated by abiotic stresses, suggesting that they may be involved in drought signaling [42-45]. Another identified gene associated to signal transduction was an ethylene-responsive factor. Ethylene is a well characterized phytohormone that may act alone or in combination with ABA in regulating gene expression under abiotic stress [46]. Calcium-transporting ATPase/calmodulin binding are also stress-signaling proteins and are responsible for regulation of the osmotic potential of the cell.

Some genes that participate in metabolism alterations as a result of the limitation caused by low levels of intracellular CO_2 _observed during drought stress were also identified only in Prata Ligeiro. Among these genes are those coding for Phosphoenolpyruvate carboxykinase, an enzyme that has a key role in nocturnal fixation of CO_2_; malato dehydrogenase, which is an enzyme particularly important for the assimilation of carbon in C4 plants; Glutamate-1-semialdehyde aminotransferase and glucose-1-fosfato adenililtransferase [47-49], both involved in carbohydrate metabolism.

It has been proposed that the mechanism involved in drought tolerance in upland rice is a result of a higher expression of genes involved in oxidative stress protection [23]. Indeed, in the present study some genes associated to the protection of the cell were expressed only in the tolerant genotype. Among them, we found a Methionine sulfoxide reductase A and a Respiratory burst oxidase homolog, which act in the recognition of reactive oxygen species (ROS) in biotic and abiotic stresses [50]. Other interesting genes identified are Metallothionein, a superfamily of low molecular weight proteins involved in metal detoxification [51] and scavenging of oxygen-free radicals, which can decrease injury in oxidative tissue, and Ferredoxin, regulated by different environmental stresses including biotic and abiotic conditions.

Genes associated to maintenance of cell turgor were also identified such as IQ calmodulin-binding and Calcium-transporting ATPase/calmodulin binding. These genes were previously reported to participate in typical defense mechanisms in upland varieties [23].

In this study we have also identified genes which have not yet been directly related to drought tolerance, such as B12Dg1 protein, Nuclear protein SET domain containing protein and Putative pollen specific protein C13 precursor, as well as genes with unknown function. Further studies need to be performed in order to assign biological function, since these genes may play important roles in plant adaptation during drought stress conditions.

#### 2.2. Drought-responsive genes identified in IRAT20

Regarding the response of the susceptible genotype to drought stress, 71 transcripts were exclusively expressed in this genotype. As in Prata Ligeiro, a high number of genes (14) with no known homologs (no hits) were identified (Table 2). Moreover, a total of 23 genes encoding hypothetical or unknown proteins were also observed. Further expression studies of these genes may reveal important genes associated to drought stress response, which have not been explored so far. This information may contribute to a better understanding of the mechanisms related to drought susceptibility in upland rice varieties.

As in Prata Ligeiro, three transcripts showed similarity to non-plant proteins and were not considered in the analysis since they probably represent contaminating sequences (Table 2). The other transcripts showed similarity to genes associated to different functions including the transport of small molecules or inorganic ions, such as HCO_3_-transporter and Vacuolar H+ pyrophosphatase. The expression of these genes was previously reported by Wang *et al*. [23] in a lowland variety. These results suggest that upland genotypes susceptible to drought may present similar responses to those of lowland varieties, which are naturally more susceptible to water deficit.

Interestingly, the well-known transcription factor WRKY was uniquely identified in IRAT20. WRKY mediates plant stress responses [52-54] and the increased expression of this protein has been frequently associated to drought stress response in rice [23,55].

### 3. Proteome analysis

**Figure 1:**
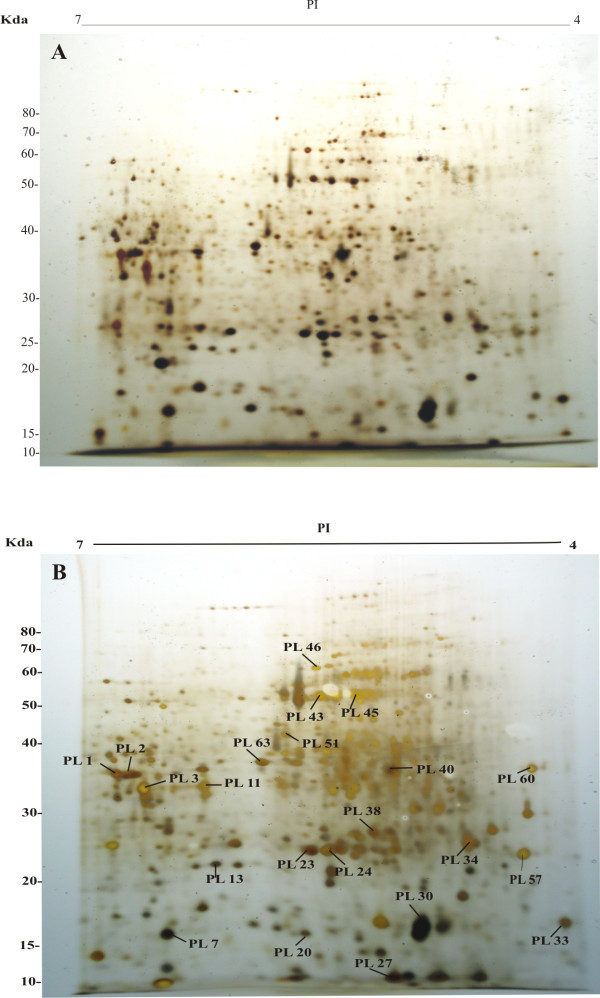
**Root protein profiles by 2-DGE of the susceptible (A) and tolerant (B) genotypes.** Total soluble protein (ca. 220 μg) was separated by 2-DGE and the spots were visualized after silver staining. Numbers indicate the protein spots successfully identified by mass spectrometry. Benchmark Protein Ladder (Invitrogen, USA) was used to estimate the molecular mass of the proteins visualized.

In order to complement the genomic studies, protein maps of roots from water-stressed plants of the susceptible (Figure 1A) and tolerant (Figure 1B) genotypes were compared. Triplicates of the gels from each genotype were compared and revealed a total of 463 proteins in the Prata Ligeiro profile and 522 in IRAT20. The two obtained synthetic gels were overlapped and this procedure allowed the identification of 307 overlapped spots, 156 proteins exclusive to the tolerant genotype and 215 proteins exclusive to the susceptible genotype. These results show a higher diversity in the protein pattern of the susceptible genotype.

**Figure 2:**
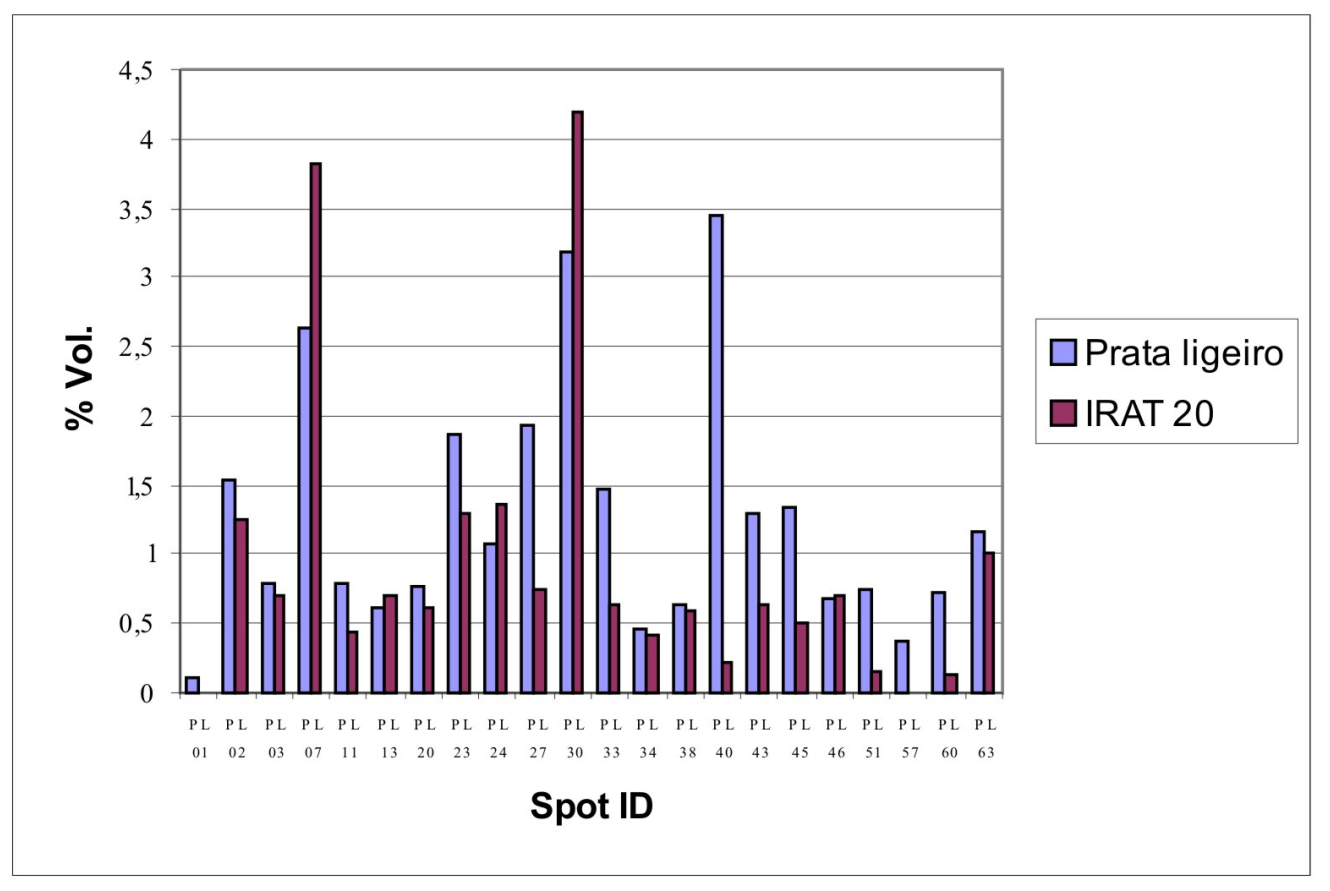
Histogram representing expression levels of up- and down-regulated proteins identified in the tolerant (Prata Ligeiro) and susceptible (IRAT20) genotypes, as determined by the Platinum software (GE Healthcare, UK).

A total of 50 intense proteins observed in the tolerant genotype profile after Coomassie blue staining was excised from the gel, digested and analyzed by mass spectrometry. By using the Mascot program, 22 proteins could be identified with a significant score (Table 3), including 16 up- and 4 down-regulated, 1 new and 1 equally expressed in both genotypes (Figure 2). The other proteins were in insufficient amounts for the identification analysis or did not return reliable matches when using the Mascot program. This probably occurs due to a low protein quantity and/or low ionization capacity of molecular components present in the samples analyzed. It is also possible that, considering the high amount of "no hits" obtained in the genomic analysis, protein sequences matching the peptides searched were not available in public databases. The peptide sequences obtained were also analyzed using the Blastp program.

**Table 3:** Proteins identified by peptide mass fingerprinting or *de novo *sequencing

**Spot n°**	**Peptide sequence**	**Protein identification**	**Accession #**	**Score**	**Mr (gel)**	**pI (gel)**	**Mr (cal)**	**pI (cal)**
PL 1		Hypothetical protein	gi|115452789	138	38.0	6,7	39	6.3
PL2		Hypothetical protein	gi|115452789	65	39.0	6.6	39	6.3
PL 3	WAPSPADAAAGR	Chitinase	gi|407472	56	36.0	6.6	35.5	7.3
PL 7	EHGAPQDENR	Zinc-superoxide dismutase	gi|22296339	26	15.0	6.4	14.7	5.9
PL 11	GPIQLSFNFNYGPAGR	Chain A, Crystal Structure Of Class I Chitinase	pdb|2DKV|A	30	37.0	6.2	32.6	5.8
PL 13	AAVGHPDTLGDCPFSQR	GSH-dependent dehydroascorbate reductase 1	gi|6939839	43	26.0	6.1	23.5	5.6
PL 20	GTSQVEGVVTLTQDDQGPTTVNVR	Putative superoxide dismutase [Cu-Zn]	gi|42408425	72	17.0	5.5	20.5	5.7
PL 23		L-ascorbate peroxidase 1,	P93404	99	28.0	5.5	27	5.4
PL 24	VATPDQAQEVHDGLR	Triosephosphate isomerase	gi|553107	49	28.0	5.4	27.5	6.6
PL 27	EFSIPLQDSGHVVGFFGR	Salt stress-induced protein	gi|158513205	88	11.0	5.0	15.1	5.1
PL 30	MIEDYLVAHPAEYA	Pathogenesis-related protein Bet v I	gi|9230755	55	18.0	4.9	16.6	4.9
PL 33	ADVGVGPVSWDDTVAAYAESYAAQR	Acidic PR-1 type pathogenesis-related protein PR-1	gi|12005673	182	17.5	4.2	17.5	4.5
PL34	WWDTFPANVDGAR	Hypothetical protein	gi|115461070	87	29.0	4.7	27.2	5.0
PL 38		Ascorbate peroxidase	NP_001060741	74	32.0	5.2	27	5.2
PL 43	MTAEIGEQVQIVGDDLLVTNPTR	Enolase	gi|780372	88	60.0	5.4	47.9	5.4
PL 46		Enolase	Q42971	74	50.0	5.4	47.9	5.4
PL 45		Hypothetical protein	gi|115465323	98	60.0	5.2	58.8	5.9
PL 51	KADATVAGDDR	Hypothetical protein	gi|125557770	37	45.0	5.7	95.7	8.0
PL 57	AGYAPPHWVQPGQGDR	Hypothetical protein	gi|125532459|	73	25.0	4.2	24.5	4.6
PL 60	ELFEQLLLHR	Chitinase	gi|561873	51	36.5	4.2	34.3	4.4
PL 63	ELVADDEWLNTEFISTVQQR	Cytosolic malate dehydrogenase	gi|115482534	66	37.5	5.9	35.5	5.75
PL 40	EFSIPLQDSGHVVGFFGR	Salt stress-induced protein	gi|158513205	104	39	5.3	15.1	5.1
		Hypothetical protein	EAY73933				40.6	8.6

Spots PL1 and PL2 (up-regulated in Prata Ligeiro) were identified as hypothetical proteins which contain Ricin B-related lectin domain. Other up-regulated hypothetical proteins were also identified and include protein spots PL34, PL45 and PL51. Spot PL45 and PL51 were expressed 2.6 and 4.5 fold, respectively, in the tolerant genotype (Figure 2), indicating that these proteins may play an important role in drought tolerance. Spot PL57 was another protein identified as hypothetical and was exclusively expressed in Prata Ligeiro. These proteins are interesting candidates for futures studies aiming at the determination of biological function.

Spots PL3 and PL60 were identified as the same protein chitinase and spot PL11 as a Chain A, Crystal Structure of Class I Chitinase. Chitinases are pathogenesis-related proteins expressed in response to biotic and abiotic stresses and have been studied in grasses such as rye in response to cold and drought stress [56]. Spot PL60 was highly induced in the tolerant genotype, which confirms the up-regulation of this protein during drought stress. Chitinases have also been reported as being induced in tomato plants tolerant to drought when compared to the susceptible genotype [57].

Two other pathogenesis-related proteins were identified: one was up-regulated (spot PL33) and the other repressed (PL30) in the tolerant genotype (Figure 2). The expression of these proteins has been previously reported in roots of rice in drought stress conditions and although the role of proteins of this family is not well established, they have been associated to hypersensitive reaction in response to biotic and abiotic factors [58]. In drought stress conditions, pathogenesis-related proteins as well as the salt stress-responsive SalT protein have been reported in rice roots [59].

As observed in the constructed cDNA libraries, several proteins involved in oxidative stress protection were induced in the tolerant genotype and were identified as a superoxide dismutase [Cu-Zn] (PL20), L- ascorbate peroxidase 1 (PL23), ascorbate peroxidase (PL38) and cytosolic malate dehydrogenase (PL63) (Table 3). Peroxidases are anti-oxidative enzymes, described in varieties of rice tolerant to high salinity conditions [25,60] and in upland rice roots in response to osmotic stress [24]. These proteins are involved in cellular detoxification and it is possible that this is a general defense mechanism in response to water deficit in upland rice. According to Wang *et al*. [23,24] tolerance to drought stress observed in upland varieties includes detoxification mechanisms, limiting the accumulation of reactive oxygen species. These authors reported that these proteins were up-regulated in upland cultivars when comparing tolerant lowland and upland rice. Unexpectedly, proteins identified as superoxide dismutase (PL7) and GSH-dependent dehydroascorbate reductase (PL13) were down-regulated in the tolerant genotype. These proteins were not identified in the genomic analysis, highlighting the importance of proteomics studies to complement the results obtained.

Another down-regulated protein (PL24) identified in the Prata Ligeiro genotype was triosephosphate isomerase (Table 3), involved in carbohydrate metabolism. According to Wang *et al*. [23], genes related to metabolism are more expressed in lowland than in upland genotypes. It is possible that susceptibility to drought in upland rice may occur in a similar way as in lowland rice.

Spots PL43 and PL46 were both identified as enolase, a glycolytic enzyme, which participates in metabolic processes. The up-regulation of enolase has been previously reported in rice roots in response to salt stress [61] and to PEG treatment [24]. Unexpectedly, PL46 was equally expressed in Prata Ligeiro and IRAT20, while spot PL43 was up-regulated in Prata Ligeiro. The existence of multiple enolase isoforms in plants has been reported [62] and it is possible that the enolases identified in this study represent different isoforms, which respond differently to drought stress conditions. Indeed, difference in the expression of enolase isoforms was observed in maize in response to anaerobiosis [63].

A highly induced protein (15 fold) in the tolerant genotype (PL40) showed identity to a hypothetical protein as well as a salt stress induced protein (Table 3). Similarly, spot 27 (2.6 fold higher in Prata Ligeiro) also presented identity to the salt stress induced protein. It is possible that these spots represent new rice proteins, not identified so far that contain a conserved region present in both matching proteins. The induction of proteins involved in tolerance to salt stress, during water deficit conditions, shows that osmotic stress is an important aspect during drought. Similar mechanisms are activated in response to different abiotic stresses, as previously reported [10].

## Conclusion

Several genes and proteins involved in drought-response as well as genes with no described homologs were identified in this work. Genes exclusively expressed in the tolerant genotype were, in general, related to maintenance of turgor and cell integrity. In contrast, in the susceptible genotype, expression of genes involved in protection against cell damage was not detected, indicating that there may be a higher degradation of cellular components in these genotypes. Similar results were obtained by Wang *et al*. [23] when comparing tolerant upland and lowland varieties. These results indicate that the mechanisms of susceptibility in upland rice are similar to those of lowland varieties, considering that the upland rice is naturally more tolerant to drought stress.

The proteomic analyses were complementary to the genomic data obtained. The expression of genes associated with cell protection against oxidative damage is considered important to cope with water deficit in upland rice. In this study, genes and proteins related to this function showed a higher expression in the tolerant genotype. Interestingly, in the proteomics analysis, the susceptible genotype showed a higher diversity in the protein profile, revealing more uniquely expressed proteins than the tolerant genotype. On the other hand, in the genomic study, the number of exclusively expressed transcripts in the susceptible genotype was lower. It is well known that transcript levels do not always reflect protein amounts [64,65]. Therefore, it is possible that the transcripts related to the proteins exclusively present in IRAT20 2D maps were in low amounts, and not detected by the genomic analysis, or they were subtracted from the control condition in the hybridization process. Differences in translation efficiency may have occurred, resulting in a higher amount of the corresponding proteins, further detected by 2-DGE. These results clearly show that proteomics studies can reveal important additional information and that the use of complementary approaches is useful for a better understanding of complex biological traits, such as drought tolerance.

Overall, due to the low amount of information regarding upland rice gene and protein expression in response to water deficit, this study sheds some light over the comprehension of this complex mechanism. However, the high amount of transcripts and proteins with unknown function obtained is still intriguing. These genes and proteins need to be further investigated in order to assign their biological function and advance our knowledge regarding drought tolerance in upland rice.

## Authors' contributions

AM, ARR, CMG, MEF and PHNR designed and performed the research. FRS analyzed the sequence data and EMS and DS analyzed the mass spectrometry data. ARR and AM drafted the manuscript. ACMB and CRS critically revised the article. All authors approved the final version.
